# Dual task gait deteriorates gait performance in cervical dystonia patients: a pilot study

**DOI:** 10.1007/s00702-021-02393-1

**Published:** 2021-07-29

**Authors:** Oscar Crisafulli, Carlo Trompetto, Luca Puce, Lucio Marinelli, Stefania Costi, Giovanni Abbruzzese, Laura Avanzino, Elisa Pelosin

**Affiliations:** 1 Department of Neuroscience, Rehabilitation, Ophthalmology, Genetics, Maternal and Child Health, University of Genoa, Genoa, Italy; 2 Ospedale Policlinico San Martino, IRCCS, Genoa, Italy; 3 Department of Surgery, Medicine, Dentistry and Morphological Sciences, University of Modena and Reggio Emilia, Modena, Italy; 4 Department of Experimental Medicine, Section of Human Physiology, University of Genoa, Genoa, Italy

**Keywords:** Cervical dystonia, Dual task, Gait, Balance, Motor-cognitive

## Abstract

Day-to-day walking-related activities frequently involve the simultaneous performance of two or more tasks (i.e., dual task). Dual task ability is influenced by higher order cognitive and cortical control mechanisms. Recently, it has been shown that the concomitant execution of an attention-demanding task affected postural control in subject with cervical dystonia (CD). However, no study has investigated whether dual tasking might deteriorate gait performance in CD patients. To investigate whether adding a concomitant motor and cognitive tasks could affect walking performance in CD subjects.17 CD patients and 19 healthy subjects (HS) participated in this pilot case–control study. Gait performance was evaluated during four walking tasks: usual, fast, cognitive dual task and obstacle negotiation. Spatiotemporal parameters, dual-task cost and coefficients of variability (CV%) were measured by GaitRite^®^ and were used to detect differences between groups. Balance performance was also assessed with Mini-BEST and Four Step Square tests. In CD participants, correlation analysis was computed between gait parameters and clinical data. Significant differences in complex gait and balance performance were found between groups. CD patients showed lower speed, longer stance time and higher CV% and dual-task cost compared to HS. In CD, altered gait parameters correlated with balance performance and were not associated with clinical features of CD. Our findings suggest that complex walking performance is impaired in patients with CD and that balance and gait deficits might be related

## Introduction

Cervical dystonia (CD) is the most common form of adult-onset idiopathic focal dystonia and is characterized by involuntary repetitive contractions of neck muscles leading to abnormal postures of the head and neck (Balint et al. 2018). Pathophysiology of CD is complex and increasing evidence highlighted that CD is related to the dysfunction of neural network that comprises the basal ganglia-cerebello-thalamo-cortical connections (Trompetto et al. 2012; Prudente et al. 2014; Brüggemann 2021).

In addition to the distinctive clinical symptoms related to dystonia, CD patients may also manifest balance and subtle gait problems that can result in reduced physical activity and fear of falling (Zetterberg et al. 2015). Postural instability has been extensively investigated in CD and results demonstrated that postural control and dynamic balance are often impaired. This is supported by the knowledge that the head posture, sensory-motor integration deficits and altered vestibular functions might affect both static and dynamic balance. Conversely, at present, few studies (Wolf et al. 2016; Barr et al. 2017; Esposito et al. 2017) examined gait performance in patients with CD. Available data show that patients walk more slowly and with an altered gait pattern: swing phase is shortened and stance phase is extended (i.e., longer stance time and double support time). In addition, results are consistent in reporting differences in gait variability compared to healthy controls. It has been hypothesized that such gait abnormalities might be related to head deviation and disturbed body-centered visual perception (Barr et al. 2017) or might depend on abnormal sensory-motor integration processes (Esposito et al. 2017).

Gait is no longer considered an automatic movement that requires only minimal cognitive resources, since a wide number of studies demonstrated that motor-cognitive reserves are essential to walk safely in everyday environments (Yogev-Seligmann et al. 2008). To date, the so-called “dual-task” (DT) walking, consisting of adding a secondary attention-demanding task during walking (e.g., walking while talking, counting or overcome an obstacle) is a simple but effective way to assess the interaction between motor and cognitive resources. Precisely, DT relies on executive functions which are needed for planning, monitoring and executing complex tasks (Hausdorff et al. 2008). Through this paradigm it is possible to measure the relative change in gait performance, known as dual-task interference or the dual-task effect, since it demands the allocation of attentional resources to the concomitant task.

In patients affected by other movement disorders, such as Parkinson’s disease (Raffegeau et al. 2019), Huntington’s diseases (Purcell et al. 2019) and Essential Tremor (Rao et al.^2013^), in which executive function impairments have been consistently shown, dual tasking has been largely investigated and results demonstrated that they are particularly susceptible to dual-task interference due to a reduction in the functional reserve that is needed for brain mechanisms involved in DT performance. Various aspects of altered cognition have been described in dystonia. For example, Romano et al. showed impairment in working memory, processing speed, visual motor ability and short-term memory in patients with CD (Romano et al. 2014).

Conversely, only two studies investigated cognitive-motor interference in CD patients (Demir et al. 2020; Baione et al. 2021). As an example, Baione et al. (2021), based on the hypothesis that balance disturbances in CD may be associated with a reduction of the attentional resources available for simultaneous performance of secondary tasks, investigated whether postural control would be worsened by a concomitant execution of attention-demanding cognitive task. Results demonstrated that in CD patients postural control deteriorates while performing a cognitive DT with further decline associated with increase task difficulty These preliminary findings support the idea that, similarly to other movement disorders, also in CD, balance problems are not entirely due to dystonic posture but are associated with a reduction of functional reserve. In the present study we want to investigate whether adding a concomitant motor and cognitive demanding task could affect walking ability in a cohort of CD patients. To this aim, we evaluated gait performance under different experimental conditions (usual walk, fast walk, dual-task, and obstacle negotiation) and we explored possible relationship with clinical features. We expect that, similar to what described for postural control under dual task, also gait will deteriorate in patients with CD for (i) increased attentional demands in CD because of changes in sensory-motor processing and/or for (ii) decrease in the functional reserve that is needed for brain mechanisms involved in DT performance because of cognitive impairments.

## Methods

### Participants

Demographic and clinical characteristics are reported in Table 1. In all, 36 participants were involved in this study. Seventeen patients (10 males, 7 females; mean age 56.41 ± 20.83 SD; range: 33–77) affected by idiopathic CD, according to published criteria (Defazio et al. 2019), were recruited from the outpatient’s clinic and nineteen healthy subjects (HS) (13 females, 6 males; mean age 50.79 ± 14.99 SD; range 31–73) were recruited from the local community as age-matched control group The following exclusion criteria were applied: (a) presence of neurological disorders other than CD or peripheral sensory neuropathy (b) sever spinal deformities or any orthopedic problems that may affect gait, (c) clinical diagnosis of dementia or other cognitive impairments (Montreal Cognitive Assessment (MoCA) score < 26) (d) presence of psychiatric abnormalities that may affect cognitive functions such as schizophrenia and major depressive disorders. This study was performed in line with the principles of the Declaration of Helsinki. Approval was granted by the Ethics Committee Regione Liguria (Protocol No. 311REG2014). All participants gave their written informed consent prior to participation.

**Table 1:** Participants characteristics

	CD (*n* = 16)	HS (*n* = 18)	Statistics
Sex M:F (*n*)	10:6	6:12	*χ*^2^ = 0.089
Age (y)	56.25 (11.85)	50.61 (10.32)	*p* = 0.15
MoCA (score)	24.33 (2.90)	27.50 (2.54)	*U* = 29.00; *p* = 0.012*
Disease duration (y)	7.3 (6.2)	–	–
TWTRS part 3 (score)	6.73 (4.93)	–	–
TWTRS total (score)	28.55 (14.44)	–	–
Mini Best (score)	23.31 (3.30)	27.33 (1.57)	*U* = 43.00; *p* < 0.001*
FSST (s)	12.20 (6.05)	8.23 (1.54)	*U* = 62.00; *p* = 0.005*

Mean (SD) are reported

*CD* cervical dystonia, *HS* healthy subjects, *M* male, *F* female, *Y* years, *MoCA* montreal cognitive assessment, *TWTRS* Toronto Western Torticollis Rating Scale, *FSST* four step square test, *S* second

*Statistical significant difference: *p* < .05

### Procedures

Participants’ testing was performed in a single day and the study protocol included two parts: (i) clinical examination and (ii) gait assessment. Participants’ characteristics (age, gender, years of education), global cognition level and general physical performances measure were obtained. The MoCA was used to assess global cognitive functions. The Toronto Western Spasmodic Torticollis Rating Scale (TWSTRS) (Boyce et al. 2012) was adopted for rating the severity of cervical dystonia.

**Fig. 1:**
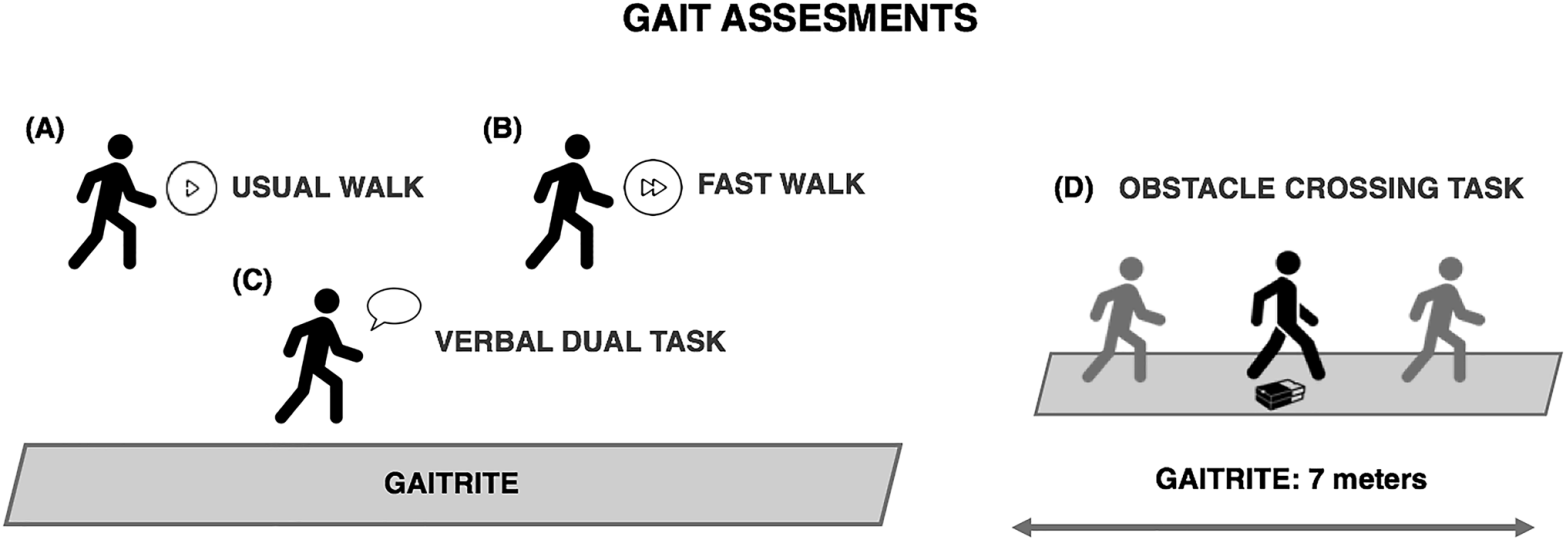
Representative image of gait assessment. The subjects were asked to walk on the GAITRite carpet *n* various conditions; (**A**) usual walk, walk to their own pace; (**B**) fast walk, walk to their maximum speed, not running; (**C**) verbal dual task, walk while saying the highest possible number of words beginning with a letter referred by the operator right before the beginning of the trial; (**D**) obstacle crossing task, walk crossing an obstacle placed 3 m from the start of the mat. The order of the task was randomly assigned

Gait tasks are depicted in Fig. 1. Gait was assessed during 4 different conditions: (I) usual walking: the subjects were asked to walk at their self-selected comfortable speed; (II) fast walking: the subjects were asked to walk to their maximum speed, not running; (III) cognitive Dual Task (DT): the subjects were asked to walk while performing a verbal fluency task; (IV) obstacle negotiation: the subjects were asked to walk while negotiating obstacles (20 cm wide × 10 cm high × 30 cm depth). The order of the gait tasks was randomized and all the subjects walked back and forth for 1 min during each task, having a 15-s rest period among the tasks. Finally, balance and dynamic stability abilities, were assessed by means of the mini-Balance Evaluation System Test (mini-BEST) (Löfgren et al. 2017) and the Four-Square Step Test (FSST) (Moore and Barker 2017) respectively. To exclude any confounding effects owing to botulinum toxin (BONT) injections, clinical and gait assessments were performed at least 4 months after the last BONT treatment.

### Gait performance

Gait parameters were measured using the GAITRite® system (GAITRite, CIR Systems, Franklin, NJ) consisting in a 7.3 m × 0.6 m long walkway with embedded pressure sensors. To ensure that steady speed walking was recorded, additional 2 m, at the beginning and at the end of the GAITRite^®^ were incorporated in each condition. For processing and data storing we used the ProtoKinetics Movement Analysis Software (PKMAS, Havertown, PA). In all the conditions the following parameters were considered: gait speed (GS), step length (SL), stance time (ST) and their coefficients of variability (CV% = SD/mean × 100). Spatiotemporal data were computed from all the steps recorded during the entire task. As previously reported (Johansson et al. 2021; Vitorio et al. 2021), to characterize the performance of usual and DT walking, the DT cost ([dual task − single task)/single task] × 100) was calculated for two key measurements (Morris et al. 1998) of walking (i.e. gait speed and stride length).

For obstacle negotiation, four additional parameters were computed: the crossing step length (i.e., the length of the step beyond the obstacle), the crossing velocity (i.e., the velocity of the step beyond the obstacle), and the trail limb position before and after the obstacle. In this task, gait parameters were calculated with respect to the planning and the execution phases of obstacle crossing. For the planning phase, spatiotemporal parameters were calculated using data derived from the last three steps before the obstacle (Pieruccini-Faria et al. 2014; Pelosin et al. 2020). For the execution phase, we evaluated the (i) crossing step length, (ii) the crossing step velocity (calculated by dividing the lead crossing stride-by-stride duration), (iii) stride length and (iv) the stride length velocity. Gait parameters were computed from all the three steps before the obstacle and all the crossing steps recorded during the entire task.

### Statistical analysis

The Chi-square test was applied to assess gender differences between groups. Prior to the analysis, all variables were examined for normality with the Shapiro–Wilk *W* test. Differences between groups (CD and HS), MoCA score, Mini-BEST score and FSST data were assessed by the non-parametric Mann–Whitney test, since data were not normally distributed. For the analysis of age and for all gait parameters recorded during usual walk, fast walk, DT, obstacle negotiation (both planning and execution phase) conditions, the comparison between CD and HS groups was performed using a un-paired *t* test. Finally, Pearson’s correlations were used to investigate any relationships between clinical (disease severity and duration), MoCA global score, balance (Mini-BEST and FSST) variables and spatiotemporal gait parameters.

Statistical analysis was performed with SPSS software (version 22). *p* values of 0.05 were considered as threshold for statistical significance. The Bonferroni correction for multiple comparisons was applied to the correlation analysis and the level of significance was adjusted to 0.05/10: i.e., *p* < 0.005.

## Results

### Participants

Participants’ characteristics and statistical analysis are reported in Table 1. Two subjects (1 CD and 1 HS) were excluded from the analysis due to data corruption during gait assessment; therefore 16 CD and 18 HS participants were included in the statistical analysis. The two groups were similar for age (*p* = 0.15) and gender distribution (*p* = 0.089), while a difference was found in the MoCA score (*U* = 29.00, *p* = 0.012).

### Gait and balance performance

Table 2 shows between-group comparisons of gait performance on the 4 walking conditions. Gait performance during usual walking task was similar between CD and HS participants (*p* always > 0.05). Conversely, participants with CD performed worse than the age-matched controls during complex walking conditions. During fast walking task, a significant difference was found for gait speed (*p* = 0.027) and gait speed CV% (*p* = 0.041), with CD patients showing a slower and more variable gait compared to HS. Stance time was longer (*p* = 0.002) with a higher CV% (*p* = 0.016) in CD subjects.

**Table 2:** Differences in performance of walking conditions between groups

Task	Variable	HS	CD	Between-groups *p* value
Usual	Gait speed (cm / s)	120.43 (15.13)	116.90 (23.78)	*p* = 0.61
	Gait speed—CV%	4.04 (0.72)	5.10 (2.83)	*p* = 0.14
	Step length (cm)	65.58 (5.58	64.23 (11.46)	*p* = 0.660
	Step length—CV%	2.96 (0.62)	3.87 (2.12)	*p* = 0.093
	Stance time (s)	0.70 (0.03)	0.75 (0.13)	*p* = 0.134
	Stance time—CV%	2.86 (0.37)	3.91 (2.28)	*p* = 0.063
Fast	Gait speed (cm / s)	174.37 (14.46)	155.96 (30.02)	*p* = 0.027*
	Gait speed—CV%	4.21 (1.38)	5.37 (1.81)	*p* = 0.044*
	Step length (cm)	77.55 (7.01)	74.05 (11.85)	*p* = 0.08
	Step length—CV%	3.01 (1.03)	3.68 (1.73)	*p* = 0.176
	Stance time (s)	0.55 (0.03)	0.60 (0.05)	*p* = 0.002*
	Stance time—CV%	3.41 (1.01)	5.27 (3.60)	*p* = 0.016*
DT	Gait speed (cm / s)	121.67 (16.97)	107.10 (22.01)	*p* = 0.036*
	Gait speed—CV%	5.77 (2.24)	8.28 (4.40)	*p* = 0.041*
	Step length (cm)	65.41 (6.26)	61.70 (8.13)	*p* = 0.384
	Step length—CV%	4.00 (1.98)	5.43 (2.85)	*p* = 0.096
	Stance time (s)	0.69 (0.06)	0.77 (0.10)	*p* = 0.022*
	Stance time—CV%	4.09 (1.51)	6.22 (3.78)	*p* = 0.035*
DT cost	Gait speed	−7.69%	1.4%	*p* = 0.030*
	Step length	−5.43%	−0.19%	*p* = 0.038*
Obstacle crossing	Stride Length	87.12 (20.02)	80.44 (20.84)	*p* = 0.348
	Stride length V	98.40 (14.00)	86.92 (16.76)	*p* = 0.037*
	Crossing step	49.37 (9.64)	44.37 (7.92)	*p* = 0.111
	Crossing step V	103.19 (17.20)	89.88 (17.72)	*p* = 0.034*

Data mean (standard deviation) for spatiotemporal parameter of gait are reported

*HS* healthy subjects, *CD* cervical dystonia, *DT* dual task, *CV%* percentage of coefficient of variability, Obstacle crossing analysis. *SL* stride length—stride before the obstacle, *SLV* stride length velocity—stride before the obstacle, *Crossing step* step crossing the obstacle, *CSV* crossing step velocity

*Statistical significant difference: *p* < .05

For DT walking, statistical analysis revealed a significant difference in gait speed (*p* = 0.036), stance time (*p* = 0.022) between the CD and HS groups. Also, CD participants showed a higher CV% in most of the parameters analyzed (i.e. gait speed, stance time and step length CVs %). Dual task costs for gait speed and step length were significantly higher in the patients with CD than in the HS.

The analysis of obstacle condition revealed that during the execution phase, the stride velocity (*p* = 0.037) and the crossing step velocity was lower in CD patients respect to HS (*p* = 0.034), whereas no difference was found for gait parameters data computed during the planning phase (*p* always > 0.05).

**Fig. 2:**
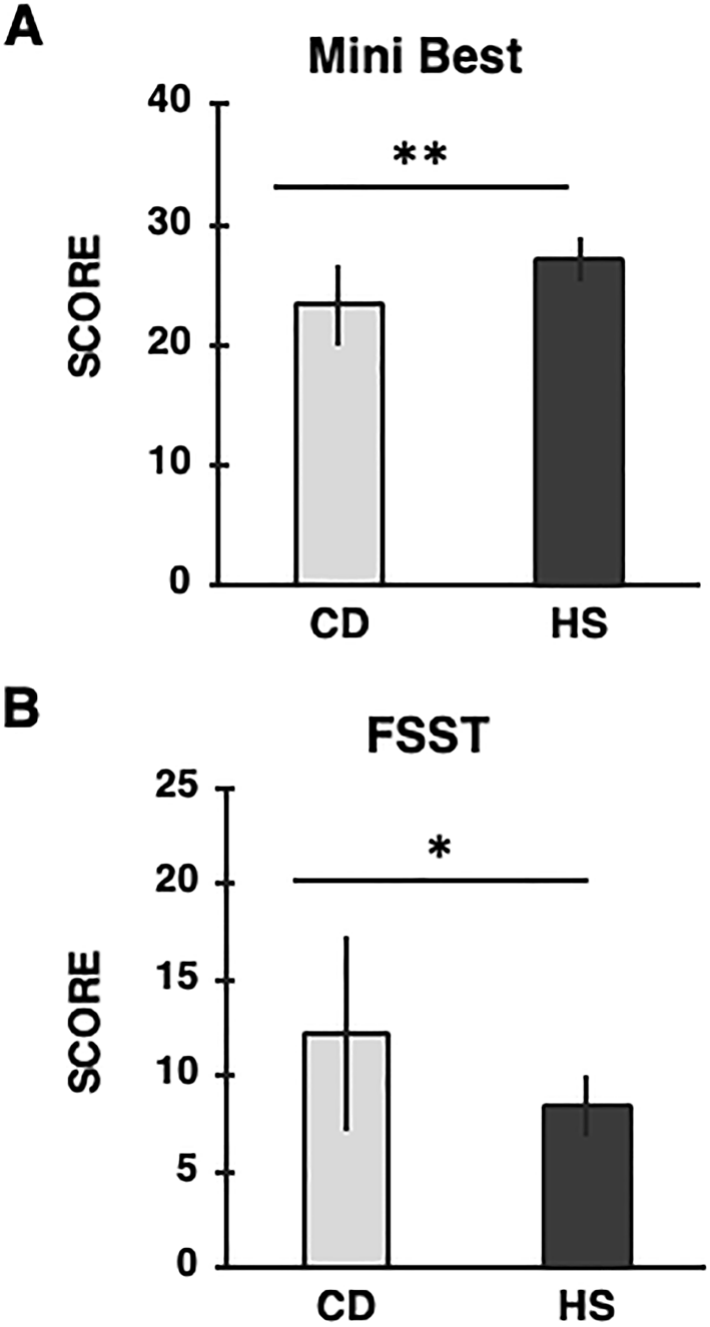
Mean values of the: (**A**) Mini Best Test (score), (**B**) Four-Square Step Test (FSST; seconds) for each group are reported. Light gray columns refer to cervical dystonia (CD) group, dark gray columns refer to healthy subjects (HS) group. Black bars represent standard deviation (SD). CD patients exhibited a lower score in the Mini Best Test and longer time to complete the FSST. Asterisks indicate significant differences between groups (*p < 0.01; ** *p* < 0.0001)

Finally, regarding balance performance (Fig. 2) CD patients exhibited a lower score in the Mini Best Test (*U* = 43.00, *p* < 0.0001) and worse results (i.e., longer time to complete the task) in the FFST (*U* = 62.00, *p* = 0.005) compared to HS.

### Correlation analysis

**Fig. 3:**
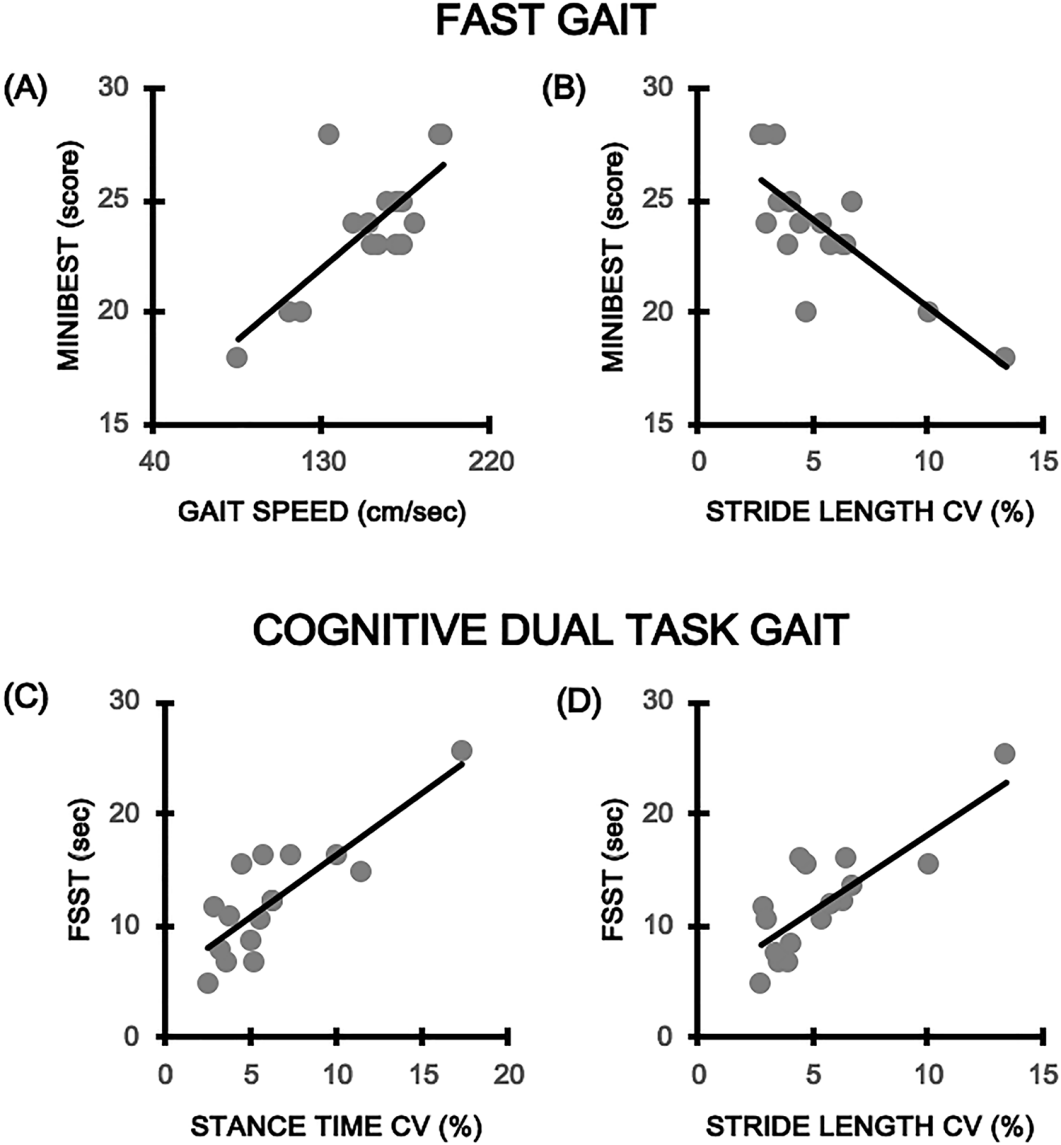
Significant correlations (after Bonferroni correction) between altered gait parameters and balance performance. Panel (**A**) and (**B**) correlation between Mini Best score (Y-axis) and gait speed and stride length CV% (X-axis) respectively. Panel (**C**) and (**D**) correlation between the FFST time (Y-axis) and stance time CV% and stride length CV% (X-axis) respectively

Results are shown in Fig. 3. Regarding FAST walking performance, among many correlations detected, only the relationship between gait speed (rho = 0.752; *p* = 0.001) and Mini-BEST score remained significant. For DT, the analysis showed a significant correlation between Mini Best and stride length CV (rho = − 0.744; *p* = 0.001) and between FSST and stance time CV (rho = 0.849; *p* = 0.000) and stride length CV (rho = 0.738; *p* = 0.001). No significant relationships were found between gait variables and disease severity (i.e., TWSTRS total score), disease duration (years) and global cognition (MoCA score) (*p* > 0.05).

## Discussion

In this study we found that complex walking is impaired in CD patients as compared to HC. Indeed, our results revealed that gait speed and step length were reduced, stance time and gait variability increased, and DT cost was higher in CD subjects during more demanding walking tasks, whereas no difference were found between groups during usual walking task. Furthermore, significant correlations were found between DT gait changes and balance impairments, but no relationships were detected with clinical features.

These results might have two possible explanations. First, reduced speed combined with increased stance time and variability is a gait pattern already observed in other neurological disorders, such as Multiple Sclerosis and Ataxia (Socie et al. 2013; Buckley et al. 2018). In the latter patients, indeed, this strategy is often used to compensate deficits in dynamic postural control by reducing the swing phase and increasing the stance phase. CD patients may adopt a similar gait pattern to compensate defective central processing of afferent information (proprioceptive feedback, vestibular inputs) (Sedov et al. 2017; Avanzino et al. 2020) thus indicating that CD patients are trying to maximize their postural control and stability during walking. Indeed, the relationship that we found between measures of balance ability and some kinematic parameters in the “dual-task” condition might suggest that patients with CD present subtle abnormalities of static balance that are effectively compensated. However, during more demanding task (such as complex gait), the coupling of all afferent information becomes functionally insufficient and gait disturbances may appear.

Second, gait deficits observed during complex gait tasks might indicate that our CD group has a decrease in the functional reserve that is needed for brain mechanisms involved in DT performance because of cognitive impairments. Indeed, global MoCA score was significantly lower in CD with respect to that of age-matched HC, indicating subtle cognitive impairments in CD participants. However correlation analysis showed no significant association between MoCA score and gait performance under dual task. The decrement in gait performance during a complex gait is largely demonstrated in the elderly (Smith et al. 2017) and several neurological diseases (Maidan et al. 2016; Belghali et al. 2017; Yang et al. 2016; Morelli and Morelli 2021). DT performance relies on the capacity to perform motor tasks automatically as well as on the cognitive (executive) ability to integrate different task demands (Strouwen et al. 2015). In idiopathic dystonia, difficulties in performing DT were firstly observed by Jahanshahi et al. (2003) where patients showed a decrement of tapping task during a concurrent task performed with the other hand. Two recent studies (Demir et al. 2020; Baione et al. 2021) also reported cognitive DT interference in CD subjects, showing a decrement of postural control during the concomitant execution of an attention-demanding tasks. In line with these results, here we found that the ability to walk while carrying out another task is impaired in CD subjects, suggesting that there are fewer attentional resources available for simultaneous performance of secondary tasks. As previously hypothesized, since recent neurophysiological and neuroimaging data supports the idea that dystonia is a network disorder (Jinnah et al. 2017; Conte et al. 2019, 2020) difficulties in performing DT could be related to impaired connectivity within different brain regions, including the basal ganglia, cerebellum, thalamus, sensorimotor and associative cortices (Avanzino et al. 2015; Bareš et al. 2019).

Nevertheless, it is important to note that, no difference emerged in usual gait performance in our cohort of CD participants compared to HCs, although previous finding reported altered gait pattern in CD (Barr et al. 2017; Esposito et al. 2017). However, also in PD and Alzheimer disease (AD) at the prodromal stage, undetectable under usual walking (i.e., self-selected speed), appear during complex gait (i.e., DT conditions) (for review see Belghali et al 2017). Mirelman et al. in healthy carriers of the LRRK2G2019S mutation (2011, 2016) showed a reduced speed, increased stride time and altered arm swing movements under DT gait performance, thus demonstrating the sensitivity of DT to unmasked compensation strategies used to counteract ganglia deficits.

As previously reported (Barr et al. 2017), in this study we found that balance and dynamic stability were impaired in CD. Indeed, our results showed that CD patients had worse scores in the Mini Best Test and took longer to perform the FSST, with an increase of the average time of about 10% compared to HS group. Abnormalities in postural control and balance were widely investigated trough different modalities. Data from instrumental assessments, showed that body sway amplitude and center of pressure displacements were enlarged in CD and that postural deficits correlated with impairments in cervical sensorimotor control, not with disease-specific characteristics, suggesting that modifications in somatosensory input from the neck or somatosensory processing might contribute to a decrease in postural control (Bove et al. 2004; Barr et al. 2017). Regarding dynamic balance, Barr et al. (2017) demonstrated that CD patients took longer time to perform the Timed Up and Go (TUG) test and that TUG impairments were associated with decrements in stepping reactions. Therefore, our results add to existing evidence that not only postural control, but also dynamic balance is compromised in CD.

Finally, in keeping with previous observations, we didn’t find any significant relationship between gait parameters and clinical characteristics of dystonia (i.e., disease severity and disease duration), supporting the idea that difficulties in performing complex walking task doesn’t strictly depend to clinical features of CD per se.

## Limitation and future directions

This study suffers some possible limitations. First of all, due to the limited sample of CD, it was not possible to establish whether gait alterations are otherwise present in the various clinical presentations of CD (type of head deviation, with or without tremor, with or without sensory trick). These aspects deserve to be studied in depth on a larger sample. Second, the lack of a comprehensive cognitive profile doesn’t allow us to determine which is the major component (motor or cognitive decline) that triggered impairments in DT performance. Indeed, although, we observed that MoCA score was significantly lower in our group of CD patients than in HS group, a structured neuropsychological evaluation for testing executive functions and attention abilities is missing. Therefore, it is difficult to disentangle what are the mechanisms behind DT impairments. Third, although patients were tested at least four months after the last BONT treatment and a specific effect of botulinum toxin on gait performance is unlikely, it would be appropriate to examine DT interference in toxin-naïve subjects in the future. Furthermore, it would be interesting in future studies assessing whether botulinum toxin injection modulates gait performance by comparing gait parmeters before and after botulinum toxin treatment.

## Conclusion

This study provides a further characterization of gait performance in CD patients and suggests that gait abnormalities come to light when a complex gait task is required. Altered gait performance under DT conditions were not related to clinical data, suggesting that they may be linked to an impaired connectivity of the networks involved in CD. Future studies are needed to confirm our results and to better determine defective mechanism underpinning dual-task interference in CD. This would open up to new therapeutic and physiotherapy interventions aimed at improving gait performance and at reducing risk of falls.

## Data Availability

Data are available upon request.
